# Reports of Societies

**Published:** 1876-05

**Authors:** 


### Reports of Societies.

### CHICAGO SOCIETY OF PHYSICIANS AND SURGEONS.

*Regular Meeting, Jan.* 10, 1876.

(Reported by EDW. WARREN SAWYER, M.D.)

Dr. Walter Hay was elected to the chair *pro tempore.*

Dr. Lenneker presented to the Society a paper on the surgical treatment of primary retro-flexion of the uterus. The first part of the paper was devoted to the clinical history and general consideration of the subject. The essayist then detailed an original operation for the relief of this displacement. An incision, some two lines in depth, is made the entire length of the internal surface of the anterior uterine wall, along the median line ; the external os uteri is cut through on each side with a pair of scissors, and the internal os incised to the depth of two lines upon each side. The edges of the incisions are kept open for a time, and the uterine cavity washed out with pure carbolic acid dissolved in alcohol. Three days after the operation the uterus is righted, and held in place by a modified Noeggerath pessary.

The incisions into the internal surface of the uterus are made with a series of three knives, devised by the author ; each knife is a blade projecting from a flexible sound-like staff. The knife intended for the anterior incision is upon the convexity of the sound ; while the blades are upon the sides of the sound in the knives intended for the lateral incisions.

The essayist has already done the operation in some twenty instances, and claims success in every instance. Moreover, he has never seen harm follow the procedure. The paper was well illustrated with drawings of instruments, position of patient, etc.

Dr. Sawyer read the notes of a case which had recently occurred in his practice : A complete placenta prævia, in a woman laboring under transitory Bright's disease; subsequent pelvic cellulitis, puerperal mania, and death on the sixteenth day; still-born fœtus, weighing thirteen pounds.

---

### CHICAGO SOCIETY OF PHYSICIANS AND SURGEONS.

*Regular Meeting, Feb.* 14, 1876.

(Reported by Edw. Warren Sawyer, M.D.)

President, Dr. Bevan, in the chair.

Dr. Chas. E. Davis reported two cases of congenital syphilis, as follows:

Mr. President and Gentlemen:

I desire to call your attention briefly to two cases of syphilis, one congenital and the other acquired, which are interesting, not only because they illustrate the remarkable tenacity of the disease in an infected constitution, but also because they exemplify a marked tendency to repair when the morbific agency is removed.

On the 19th of November, 1875, Mrs. G—— brought her child to me, a male infant four months old, having two large and scaly ulcers upon its face, one upon the forehead, the other covering the lower third of the nose, involving both nostrils and well nigh occluding them. Another large ulcer existed upon the left parietal eminence. With one exception, all the finger and toe nails had sloughed off, the matrix in several cases being the seat of dirty, greyish ulcers, and the extremity of each digit being clubbed. The skin was of a dull, coppery color, the eyes watery and of a dull, leaden hue. There were small blisters on the soles of the feet and also on the lips.

The superficial veins of the head were much enlarged, varicose and filled with dark blood, which presented a remarkable contrast with the dry and hairless scalp. The cervical and axillary glands were slightly enlarged, and the fauces congested but apparently not ulcerated.

On various parts of the body were dark patches which the mother said were the sites of former ulcers similar to those on the face.

The child had been born prematurely, and at birth was puny, cross and feeble. In 24 hours it had become covered with scarlet patches.

The mother seemed ignorant of the nature of the disease affecting her infant, and, in answer to my questions, affirmed that she had not suffered from any eruption, swollen glands or other evidences of disease for years. She admitted, however, that soon after her marriage, three years before, she had had some "soreness of the groin," for which she had received some medicine from her husband, but that she had since remained perfectly well. She had had two abortions, one at 7 months and one at 6 months, the causes of which she did not know. Neither fœtus had been seen by her.

Her husband, whom I had an opportunity of examining, was a robust and healthy looking man. He admitted that he had suffered from syphilis seven years before, for which he had been at once treated, and from which he had not since suffered.

The child was placed upon a careful hygienic regimen, Nov. 19, 1875, and the following alterative diuretic in half-teaspoonful doses, three times daily, was administered in sweetened water: ℞. Potass. bromid. ʒi; Syr. Stillingiæ Co. ʒij; Spts. nitr. dulc. ℥ss; Aq. fluv. ad ℥iv. M.

After the first day, I ordered one-eighth of a grain of calomel in one grain and one-eighth of sugar of milk, to be given at noon in place of the diuretic.

Nov. 21. The color of the skin somewhat improved. The urine yellow, profuse and offensive. Two powders daily, and one dose of the diuretic.

Nov. 23. Marked improvement in the color and condition of the ulcers; slight feverishness, restlessness and parched lips. Medicine discontinued for two doses.

Dec. 1. The ulcers quite healed; the skin clean; the eyes bright; all signs of proper nutrition returning. No further unpleasant effects from the medicine, which was ordered to be continued unless some disagreeable effect was produced, and then omitted.

Dec. 4. The child was suffering from a severe cold and had not taken medicine for one week.

Three small patches were then visible on the head, which slowly deepened in color. The medicine was then resumed with the improvement which you can notice. The hair which you see on the scalp, appeared three weeks ago.

(The infant was presented to the Society.)

The points of interest in this case, are:

1. That an infant so diseased should survive.

2. That, under any treatment, its feeble organism should be able to repair such extensive lesions in ten days.

3. That toleration of so extended a course of medication should be established in so young an infant.

The second case I report, is that of the nurse of this child, and it illustrates the fact that an infected individual may remain free from external evidences of the disease for months, or until some intercurrent disease arouses it to an activity which seems the more energetic for its period of incubation.

When I first saw Mary McM—, she was a remarkably healthy-looking and robust Irish servant girl, with a clear complexion, of good habits, modest deportment and an unblemished moral reputation. I was called to attend her Nov. 20, 1875, for a severe cold with menstrual suppression, resulting from washing some stone steps while suffering from the periodic discharge. There was some ulceration of the fauces which proved persistent, but which, considering the character of the girl, did not awaken my suspicions. The menstrual flow was not restored. Soon after, Dec. 29, 1875, she came to consult me for a "queer heat" brought out by some warm baths. I at once recognized the syphilitic nature of this eruption, and elicited the following facts: She had come from Canada during the preceding June, and was sent by a charitable institution to nurse the mother of the infant whose case is detailed above, during her confinement. She was thus engaged during seven weeks, two before and five after that event. She states that her sympathies having been greatly excited by the condition of the puny infant, she had taken it to bed with her, kissed it and soothed it when the mother was incapable of rendering it such services. This was admitted before she was aware of the nature of the malady with which she was afflicted.

This patient was transferred to the Women's and Children's Hospital, Dec. 29, 1875, where she was treated with alteratives and mercurial inunction for eight days with decided benefit, but was then made an out patient in order to make room for other applicants. She was then transferred to the County Hospital where she suffered (Feb. 12, 1876,) with some intercurrent inflammatory disorder of the bowels. Convalescence began on the 27th day of February, 1876. On the 6th of March, 1876, she was able to sit up in bed, and the syphilitic manifestations upon the surface of the skin were rapidly disappearing.

In commenting upon these cases, Dr. Hyde remarked, that it would be interesting to ascertain the exact facts respecting the syphilitic history of the father, the syphilitic infection of the mother, the site of inoculation of the nurse, and the precise sequence of events in the case of the latter. It is an interesting question which is variously answered, whether it is possible for a syphilitic father to beget a syphilitic child without the intervention of the disease in the mother. A syphilitic father when the mother is free from the disease, rarely, if ever, begets a syphilitic child, the popular idea upon this subject being greatly at fault. A child congenitally syphilitic argued a syphilitic mother. Whether the influence of the mother is transmitted to her offspring merely through the medium of the blood, or whether the characteristic lesions of the child syphilitic at birth are largely due to the degenerative changes in the placenta which have lately attracted the attention of eminent obstetricians as well as syphilographers, are also questions of interest.

There are two classes of congenitally syphilitic children, the curable and the incurable; the latter, surviving the dangers of syphilitic fœtal life, soon succumb. The former class are best treated by mercurial inunction, the medicament being applied to the swathing bands of the infant.

Respecting the danger to the practitioner referred to by the reporter as occurring in the management of syphilitic patients, Dr. Hyde remarked further, that it was a significant fact, that the large proportion of medical men thus infected, are either of uncleanly habits or were accidentally inoculated while making the vaginal touch, the finger thus situated being bathed with the moist secretions of the part and being subjected to a high temperature admirably calculated to favor the contagious process. Under such circumstances the cutaneous integument of the finger becomes a quasi-mucous surface, and, as such, incurs the dangers of exposed mucous surfaces.

---

### THE CHICAGO MEDICAL SOCIETY.

*Meeting March* 6, 1876.

Dr. N. Bridge reported a case of perityphlitis in a female, aged about forty, where the disease had existed for three or four weeks, following obstinate constipation and impaction of fecal matter in the cæcum and ascending colon. The bowels had been emptied early in the progress of the case by cathartics and injections—several soft bilious dejections following a larger discharge of hard scybalous masses, and the tumefaction in the ascending colon disappearing. There was a slight tumefaction in the ileo-cæcal region that was exceedingly tender to the touch, and after each movement of the bowels was painful often for several hours. Pain was produced in this part and in the groin on flexing the right thigh ; at times it was quite impossible to move the thigh. Late in the case, the glands in the groin became slightly enlarged and very tender. The temperature had been one to two degrees (F.) above the normal in the morning, with an increase of one to two degrees in the evening throughout, and the pulse had ranged from 75 to 110. The tongue had been intensely red early, but becoming less red and more moist toward the termination of the case.

There were during much of the time night sweats, and the general prostration was considerable. Although a small quantity of purulent matter was discharged from the vagina, it could not be demonstrated by examination as coming from the point of inflammation.

Two weeks after the bowels were emptied, or supposed to be, there occurred half a dozen discharges, in three or four days, of large, hard, and, many of them, very dry masses of fecal matter in large quantity. These discharges were, in most instances, induced by injections and small doses of castor oil. Following them the patient speedily got well. She had eaten little or nothing but milk and broths. The question of the age of the feces discharged during the convalescence was an interesting one. Were they in part the material of the old impaction, or had they been formed during the sickness? Dr. B. had only seen one of the dejections ; this one was clearly recent in its formation, but the descriptions of others given by the patient and her husband would suggest the thought that, because of their dryness and hardness, they might have been incarcerated in the bowels from the beginning of the ailment.

In either case he was confident the soft fecal discharges which occurred during the first week of the case, and following that of scybalæ, had come from the small intestines and *above* the seat of the accumulation in the caput coli and ascending colon.

Dr. H. M. Lyman had seen the case in consultation, and thought there was a slight grade of general inflammation in the intestinal walls, as well as that localized in the neighborhood of the ileo-cæcal region.

In the discussion of the case, Dr. Lyman believed it perfectly supposable that the fecal matter finally discharged had been formed during the existence of the inflammation. Intestinal contents in contact with inflamed mucous membrane, often become very dry and hard in a surprisingly short time. He thought this fact was not sufficiently borne in mind ; forgetting it, practitioners had been led to suppose that specimens of fecal matter —grown hard and dry in contact with inflammation— only a few days old, had been impacted in the abdomen as many weeks or months.

---

### ROCK RIVER MEDICAL SOCIETY.

(Reported by F. E. Sherman, M.D.)

The Society convened in Hartford, Nov. 20, pursuant to adjournment. President Senn in the chair.

Dr. Lynch read an essay entitled "The Relation of the Heart's Action to the Nervous System and to Mental and Emotional States." This was followed by the presentation of the following case : Mr. A— G—, æt. 36, farmer ; married ; of temperate habits ; never had had any severe illness. About two years ago, he noticed a sensation of numbness and pricking in the right upper extremity, which in a few weeks subsided and appeared in the feet, gradually extending upward until the hips were affected. After this his condition remained unchanged to the present time. He now finds it difficult to keep the lower extremities warm, and tires easily on walking ; the muscles feel sore, but he walks naturally without loss of co-ordination. The abdominal and thoracic viscera are in a healthy condition and their functions performed in a natural manner. He has not been addicted to excess of venery, but there is a partial loss of desire. The battery indicates loss of sensory but normal motor power. On applying compresses he perceived two points $3\frac{1}{16}$ inches apart, on the outer side of the left leg; $\frac{3}{4}$ inch on the inner side. On the outer side of the right leg, $7\frac{3}{16}$ inches; inner side 2 inches. It was thought to be due to atony of the nerves from mechanical compression by a morbid growth or to chronic spinal meningitis.

The regular discussion related to the Duties of a Physician in Natural Labor.

The next meeting will be held at West Bend, Dec. 4. Subject for discussion: "Non-inflammatory Softening of the Spinal Cord."

---

### THE NORTH CENTRAL MEDICAL ASSOCIATION.

(Reported by F. COLE, Sec.)

This Association, comprising the counties of Woodford, Marshall, Putnam, Livingston and La Salle, held its annual meeting at Wenona, Tuesday, December 7th. Dr. E. P. Cook, of Mendota, President, in the chair.

Drs. D. E. Thomas, of Lacon, and J. W. Evans, of Varna, were elected members.

The following reports were made: "The Germ Theory"—Dr. D. W. Lamme, Eureka.

"Differential Diagnosis"—Drs. A. Reynolds, El Paso, and Wm. O. Ensign, New Rutland.

"New Remedies"—Drs. G. F. Roberts, Lacon, and J. S. Whitmire, Metamora.

"Improvements in Surgery"—Dr. F. Cole, El Paso.

"Gynecology"—Dr. E. P. Cook, Mendota.

The following officers were elected for the ensuing year: Drs. I. H. Reeder, Lacon, *President;* A. Reynolds, El Paso, *Vice-President;* F. Cole, El Paso, *Sec'y and Treasurer.*

The president appointed the following committees to report at the next meeting:

"Bloodletting in Acute Inflammation"—Drs. J. S. Whitmire, Wm. O. Ensign, A. Reynolds.

"Plaster of Paris in the Treatment of Fractures"—Drs. I. H. Reeder, D. E. Thomas, Enoch Blanchard.

"The Study of the Natural History of Disease"—Drs. G. Newkirk, G. F. Roberts, J. W. Evans.

"The Best Means to Encourage and Sustain Local Societies"—Drs. F. Cole, L. G. Thompson, W. L. Downey.

Resolutions were passed, asking that local and State medical societies encourage a higher standard of medical education. Also, that Drs. Whitmire and Roberts be requested to publish the portions of their papers pertaining to original research with New Remedies.

The following delegates were appointed:

To State Medical Society—Drs. E. Blanchard, Minonk; I. H. Reeder, Lacon; D. W. Lamme, Eureka; Wm. O. Ensign, New Rutland.

To American Medical Association—Dr. F. Cole, El Paso.

Adjourned to meet at the same place in one year.

